# Harnessing the strategy of metagenomics for exploring the intestinal microecology of sable (*Martes zibellina*), the national first-level protected animal

**DOI:** 10.1186/s13568-020-01103-6

**Published:** 2020-09-18

**Authors:** Jiakuo Yan, Xiaoyang Wu, Jun Chen, Yao Chen, Honghai Zhang

**Affiliations:** 1 Qufu Normal University, Qufu, 273165 China; 2 College of Marine Life Science, Ocean University of China, Qingdao, Shangdong China

**Keywords:** Sable (*Martes zibellina*), Metagenomics, Gut microbiota, Functional database, Gene function annotation

## Abstract

Sable (*Martes zibellina*), a member of family *Mustelidae*, order *Carnivora*, is primarily distributed in the cold northern zone of Eurasia. The purpose of this study was to explore the intestinal flora of the sable by metagenomic library-based techniques. Libraries were sequenced on an Illumina HiSeq 4000 instrument. The effective sequencing data of each sample was above 6000 M, and the ratio of clean reads to raw reads was over 98%. The total ORF length was approximately 603,031, equivalent to 347.36 Mbp. We investigated gene functions with the KEGG database and identified 7140 KEGG ortholog (KO) groups comprising 129,788 genes across all of the samples. We selected a subset of genes with the highest abundances to construct cluster heat maps. From the results of the KEGG metabolic pathway annotations, we acquired information on gene functions, as represented by the categories of metabolism, environmental information processing, genetic information processing, cellular processes and organismal systems. We then investigated gene function with the CAZy database and identified functional carbohydrate hydrolases corresponding to genes in the intestinal microorganisms of sable. This finding is consistent with the fact that the sable is adapted to cold environments and requires a large amount of energy to maintain its metabolic activity. We also investigated gene functions with the eggNOG database; the main functions of genes included gene duplication, recombination and repair, transport and metabolism of amino acids, and transport and metabolism of carbohydrates. In this study, we attempted to identify the complex structure of the microbial population of sable based on metagenomic sequencing methods, which use whole metagenomic data, and to map the obtained sequences to known genes or pathways in existing databases, such as CAZy, KEGG, and eggNOG. We then explored the genetic composition and functional diversity of the microbial community based on the mapped functional categories.

## Introduction

The communities of microorganisms residing in the gastrointestinal (GI) tract of animals are vast and diverse, with cell numbers exceeding the number of host cells (Krone et al. 2014; Li et al. 2018a, b; Zhu et al. 2018). The intestinal microbial population can be considered a separate organ that encodes 150-fold more genes than the host genome (Guan et al. 2016; Hasan et al. 2019; Jain et al. 2018). In general, the gut microflora is considered a diverse and dynamic ecosystem that maintains the homeostasis of the intestinal tract (Ma et al. 2019; Oliphant and Allen-Vercoe 2019; Taha-Abdelaziz et al. 2018). The status of the intestinal microbiota is closely related to intrinsic and extrinsic host factors, including birth, diet, nutrition, stress, drugs, habitat and social contact (Hale et al. 2019; Jang et al. 2018; Li et al. 2017; Robertson et al. 2018). Gut microorganisms are indispensable for nutrient absorption by the host and host metabolism (De Mandal et al. 2018; Deng et al. 2019; Dong et al. 2018; Pan et al. 2019; Si et al. 2020). For instance, *Firmicutes*, *Bifidobacterium*, and *Lactobacillus* have multiple beneficial effects on host metabolism, producing energy and short-chain fatty acids (SCFAs) (Antunes et al. 2019; Bang et al. 2018; Blakeley-Ruiz et al. 2019). Furthermore, the intestinal microbiota regulates carbohydrate and lipid metabolism (Li et al. 2018a, b; Pekkala et al. 2017), providing the host with sources of energy or activating receptors (Federici 2019). Intestinal microorganisms are also involved in the synthesis of key vitamins that cannot be produced by their host organisms (Grieneisen et al. 2019; Martin et al. 2018; Srugo et al. 2019). Accumulating evidence shows that antigenic stimuli from gut microbiota play significant roles in shaping the intestinal immune responses that can affect host health (Doulberis et al. 2015; Liu et al. 2019; Wu et al. 2017a, b; Xue et al. 2019). The intestinal bacteria also influence the development of the intestinal epithelium to strengthen intestinal barrier function (Hill et al. 2016; Li et al. 2019; Xu et al. 2020).

The sable (*Martes zibellina*), a carnivorous mammal distributed across the cold northern zone of Eurasia (Li et al. 2013), is famous for its valuable, warm fur (Guan et al. 2016; Monakhov et al. 2018; Svishcheva and Kashtanov 2011). The sable has a slender body and a high surface-to-volume ratio, and it maintains a low body fat percentage of approximately 8%, even during particularly cold winters (Mustonen and Nieminen 2006). Thus, a high metabolic rate should be required for its survival (Mustonen et al. 2006). We sought to investigate the correlations of metabolic functions with the intestinal flora of sable. Traditionally, the type of intestinal microflora has been determined through bacterial culture (Wu et al. 2017a, b). However, the growth environments of many bacteria cannot be adequately replicated in vitro, which hinders the study of microbial diversity and function (Tully et al. 2018). In recent years, the progress of high-throughput sequencing technologies has allowed fundamental advancements in DNA sequencing (Shui et al. 2020). With the declining expense of DNA sequencing, metagenomics has been rapidly developing (Johnson 2019). Metagenomics techniques are commonly used to sequence and analyse the whole genomes of microbes from a sample without the need for cell culture. Metagenomics is widely applied to probe environmental microbial diversity at multiple levels and enables the study of microbial community structure and ecosystem function (Martin et al. 2018).

In this study, we sought to identify the complex structure of the intestinal microbial population of sable based on metagenomic sequencing methods, which use whole metagenomic data, and to map the obtained sequences to known genes or pathways in existing databases, such as CAZy, KEGG, and eggNOG. We then explored the genetic composition and functional diversity of the microbial community based on the mapped functional categories. In ancient China, the sable was considered a valuable fur animal; however, at present, there is no mature rearing strategy. The present work was devoted to acquiring a detailed view of the functional structure of the intestinal flora, and it provides valuable information that can guide sable breeding.

## Materials and methods

All faecal samples analysed in this study were collected at Dalian Mingwei Marten Industry Company Limited and derived from wild sables imported from Mohe County of Heilongjiang Province and Greater Khingan Range. As carnivores, the sables were fed fish and chicken. When collecting stool samples, we recorded information on host gender, sampling date and cage number. Fresh faecal samples from sables were collected aseptically in sterile stool containers and immediately frozen in a freezer. The samples were categorized into three groups, with the five faecal samples from female sables labelled MZF.1–MZF.5, the four faecal samples from male sables labelled MZM.1–MZM.4, and the samples of intestinal contents labelled MZS.1, MZB.1 and MZB.2 (Table 1). Before DNA extraction from the faecal samples, we ensured the samples were used immediately upon removal from the freezer and avoided sample contamination.

**Table 1:** Table of the information in samples

Species	Sample	Sex	Time
Sable	MZF.1	Female	2017.11
Sable	MZF.2	Female	2017.11
Sable	MZF.3	Female	2017.11
Sable	MZF.4	Female	2017.11
Sable	MZF.5	Female	2017.11
Sable	MZM.1	Male	2017.11
Sable	MZM.2	Male	2017.11
Sable	MZM.3	Male	2017.11
Sable	MZM.4	Male	2017.11
Sable	MZS.1	Male	2017.11
Sable	MZB.1	Male	2017.11
Sable	MZB.2	Male	2017.11

### DNA extraction, library preparation and metagenomics sequencing

The QIAamp Fast DNA Stool Mini Kit (Qiagen, Hilden, Germany) was used to extract microbial DNA from the sable stool samples. Before library construction, the DNA was evaluated for quality control and quantified. Agarose gel electrophoresis (AGE) was used to analyse the purity and integrity of the DNA, and Qubit 2.0 (Invitrogen, USA) was used to precisely quantify DNA concentration. During library construction, qualified DNA samples were randomly broken into fragments approximately 350 bp in length with an ultrasonic crusher (Covaris, UK). Then, the fragments were end-repaired, A-tailed, and ligated to adapters. After library preparation, Qubit 2.0 (Invitrogen, USA) was used for initial quantification, and the library was diluted to 2 ng/µl. Subsequently, an Agilent 2100 Bioanalyzer (Agilent, USA) was used to determine whether the insert sizes of the library corresponded to expectations. To ensure library quality, real-time q-PCR was used to accurately quantify the effective concentration (> 3 nM) of the library. After the library passed the inspection, sequencing was implemented on an Illumina HiSeq X Ten platform (Illumina, USA). The raw reads are available at the NCBI Sequence Read Archive (BioProject ID PRJNA630144, SRA SRP265006).

### Quality control and genome assembly

In metagenomics research, the raw genome data obtained after sequencing include adapter information and low-quality bases, which interfere with subsequent analysis. Therefore, the raw data require quality control to remove interfering data and obtain clean data. Because of the possibility of host genome contamination, we searched the data against a database of host genes to filter out reads from host genes (SOAP aligner parameter settings: identity ≥ 90%, -l 30, -v 7, -M 4, -m 200, -×400). Reads with a quality value less than 38 (different from the default setting of ≤ 40) and number of Ns (undetected bases) at or exceeding the set number (default set to 10) were removed. In addition, reads with an overlap between the adapter and the sequence exceeding a certain threshold (≥ 15 bp) were removed. Clean data were obtained after these filtering steps, and SOAP denovo assembly software was used for assembly analysis (Luo et al. 2012). For each sample, k-mer = 55 was selected to obtain the assembly results (assembly parameters: -d 1, -M 3, -R, -u, -F) (Brum et al. 2015; Feng et al. 2015; Qin et al. 2014; Scher et al. 2013). The scaffolds were interrupted from the N-junctions to obtain N-free sequence fragments called scaftigs (i.e., continuous sequences within scaffolds) (Mende et al. 2012). The clean data for each sample were compared to the scaftigs of each sample by SOAP aligner software to obtain PE reads (alignment parameters: -u, -2, -m 200). After pooling the clean reads from each sample, k-mer = 55 was selected for mixed assembly (Karlsson et al. 2013), the remaining assembly parameters were the same as those used for single sample assembly. The scaffolds were broken from the N-junctions to obtain scaftig sequences without Ns. For the scaftigs generated by both single sample assembly and mixed assembly, fragments less than 500 bp were filtered out (Zeller et al. 2014), and statistical analysis and subsequent gene prediction were performed.

### Gene prediction and abundance analysis

Employing the scaftigs for each sample assembly and mixed assembly ( > = 500 bp), MetaGeneMark was used for open reading frame (ORF) prediction (Li et al. 2014). Fragments less than 100 nt in length were filtered out from the prediction results. For the ORF prediction results of each sample, CD-HIT software was used to remove redundancies, yielding an initial non-redundant gene catalogue. Clustering was conducted with identity 95% and coverage 90%, and the longest sequence was selected as the representative sequence (parameters: -c 0.95, -G 0, -aS 0.9, -g 1, -d 0). The clean data for each sample were compared with the initial gene catalogue by SOAPaligner, and the number of reads of genes in each sample was calculated (alignment parameters: -m 200, -×400, identity ≥ 95%). The number of genes supporting reads in each sample ≤ 2 were filtered out to obtain the final gene catalogue for subsequent analysis (Qin et al. 2012). The abundance information of each gene in each sample was calculated from the number of reads and gene length (Villar et al. 2015). Based on the abundance information of each gene in each sample, descriptive statistics were calculated, and core-pan gene analysis, sample correlation analysis, and Venn diagram analysis of gene number were conducted (blastp, evalue ≤ 1e−5).

### Species annotation

DIAMOND software was used to compare the unigenes with the sequences of bacteria, fungi, archaea and viruses extracted from the NCBI NR database (Buchfink et al. 2015). Alignment filtering was conducted using evalue ≤ minimum evalue * 10 for each sequence alignment for subsequent analysis. Multiple alignment results for each sequence may arise after filtering, yielding different species classification information. Thus, to ensure its biological significance, the LCA algorithm (a systematic classification algorithm applied in MEGAN software) was adopted to assign the classification level before the first branch as the species annotation information of the sequence (Huson et al. 2011). Based on the LCA annotation results and the gene abundance table, the abundance information of each sample at each classification level (genus and species) was obtained. The abundance of a species in a sample was determined as the sum of the gene abundance of the species annotated. For each species, the number of genes in a sample was equal to the number of genes with abundances greater than 0 in the annotated species.

### Functional database and resistance gene annotation

Currently, the commonly used databases providing functional annotations mainly include Kyoto Encyclopedia of Genes and Genomes (KEGG), Evolutionary Genealogy of Genes: Non-supervised Orthologous Groups (eggNOG), and Carbohydrate-Active Enzymes Database (CAZy). The KEGG database was introduced by Kanehisa Laboratories in 1995 with version 0.1. It has since developed into a comprehensive database, the core of which is the KEGG pathway database and the KEGG Ortholog database. In the KEGG Ortholog database, genes performing the same function are clustered together into groups called ortholog groups (KO entries). In the KEGG pathway database, biological metabolic pathways are divided into 6 categories: cellular processes, environmental information processing, genetic information processing, human diseases, metabolism, and organismal systems. The second layer currently includes 43 seed pathways, the third layer comprises metabolic pathway diagrams, and the fourth layer provides specific annotation information for each metabolic pathway map. The eggNOG database uses the Smith-Waterman comparison algorithm to annotate the orthologous groups of genes. eggNOG V4.1 covers 2,031 species and approximately 190,000 orthologous groups. The CAZy database is used to annotate carbohydrate enzymes and covers six main functional categories: GHs (glycoside hydrolases), GTs (glycosyl transferases), PLs (polysaccharide lyases), CEs (carbohydrate esterases), AAs (auxiliary activities) and CBMs (carbohydrate-binding modules). DIAMOND software was used to compare unigenes with each functional database (blastp, evalue ≤ 1e−5). In the alignment filtering step, the alignment results of each sequence with the highest score (one HSP > 60 bits) were selected for subsequent analysis. Based on the results of the functional annotations and the gene abundance table, the number of genes in each sample at each classification level was obtained. The number of genes with a certain function in a sample was calculated as the number of genes with non-zero abundance. Based on the abundance table at each classification level, analyses of the number and relative abundance of annotated genes were conducted. Resistance Gene Identifier (RGI) software provided by CARD was employed to compare unigenes with the CARD database (RGI built-in blastp, evalue ≤ 1e–30) (Qin et al. 2010). Based on the comparison results of RGI and the abundance information of unigenes, the relative abundance of ARO was calculated. Employing the ARO abundance data, a Venn diagram of abundance distribution was constructed, ARO differences between groups were analysed, and species attribution analysis of resistance genes (with focus on ARO unigenes) was conducted.

## Results

### Extraction of total microbial DNA from samples

The microbial genomes of the samples were extracted using the QIAGEN kit specialized for DNA extraction from stool samples. Total DNA was preliminarily detected by agarose-gel electrophoresis, and the total DNA concentration was detected by Qubit fluorometer to determine whether the samples met the requirements for database construction. The results are shown in Table 2.

**Table 2:** The detection report of DNA

Sample name	Concentration (ng/µl)	Volume (µl)	Total (ng)	Library type	Test results
MZF.1	11.4	51	581	Meta	A
MZF.2	13	51	663	Meta	A
MZF.3	7.6	51	388	Meta	A
MZF.4	10	51	510	Meta	A
MZF.5	0.8	51	41	Meta	A
MZM.1	37	51	1887	Meta	A
MZM.2	5.7	51	291	Meta	A
MZM.3	16	51	816	Meta	A
MZM.4	2.54	51	130	Meta	A
MZS.1	7.2	51	367	Meta	A
MZB.1	4.7	51	240	Meta	A
MZB.2	21	51	1071	Meta	A

### Sequencing data statistics

The Illumina HiSeq 4000 sequencing platform was used to obtain the original data (raw data), and the sequencing data were statistically analysed. For quality control, low-quality reads were removed. In total, after size filtering and quality control, read numbers were obtained. The clean data accounted for more than 98% of the raw data, showing that the data met the quality requirements for subsequent analysis. Descriptive statistics are shown in Table 3.

**Table 3:** The statistical information of sample data

Sample	Raw data	Clean data	Clean_Q20	Clean_Q30	Clean_GC (%)	Effective (%)
MZF.1	6423.43	6348.62	97.36	95.16	52.27	98.835
MZF.2	6400.51	371.69	97.28	95.07	49.81	99.55
MZF.3	6337.84	6290.67	97.46	95.33	51.35	99.256
MZF.4	6792.60	6769.70	97.12	94.95	41.59	99.663
MZF.5	6390.60	6341.12	96.89	95.33	45.32	99.226
MZM.1	6272.72	6260.54	97.11	94.76	47.25	99.806
MZM.2	7024.25	7010.36	96.72	94.53	38.97	99.802
MZM.3	6252.62	6230.76	97.25	95.01	50.56	99.65
MZM.4	6612.68	6556.58	95.95	94.00	41.27	99.152
MZS.1	6438.21	6360.45	96.71	94.22	42.41	98.792
MZB.1	6266.57	6244.48	96.58	94.82	47.00	99.648
MZB.2	6809.10	6693.22	96.75	91.59	47.48	98.298

### Valid data assembly results

After quality control and filtering, the data were assembled. After determining the overlap between sequences based on sequence similarity, we constructed contig sequences. Furthermore, scaffold sequences were obtained by connecting contig sequences based on paired-end relationships. The scaffold sequences contained gaps, denoted by N or n. Next, the assembled scaffolds were interrupted from the N connections to obtain scaftig sequence fragments without N. We used NOVO_MIX to filter out the scaftig fragments under 500 bp. The remaining sequences were used for subsequent analysis. The results of data assembly are shown in Table 4.

**Table 4:** The statistical information of sample assembled results

Sample	Total len. (bp)	Num.	Average len. (bp)	N50 Len. (bp)	Max len. (bp)
MZF.1	36,356,589	34,785	1045.18	1087	73,452
MZF.2	31,081,373	22,094	1406.78	1711	305,593
MZF.3	18,869,947	14,803	1274.74	1492	39,052
MZF.4	62,710,239	46,632	1344.79	1670	91,860
MZF.5	32,307,271	23,551	1,371.80	1703	52,978
MZM.1	45,524,309	37,738	1206.33	1328	226,903
MZM.2	79,892,828	65,995	1210.59	1373	185,211
MZM.3	14,205,478	11,729	1211.14	1341	41,989
MZM.4	33,869,693	26,595	1273.54	1511	86,091
MZS.1	33,880,650	26,234	1291.48	1419	243,912
MZB.1	30,495,188	17,768	1716.30	2789	400,796
MZB.2	2,101,244	2820	745.12	692	15,984
NOVO_MIX	205,647	273	753.29	700	5562

### Gene prediction

After data assembly, MetaGeneMark was used to predict the open reading frames (ORFs). The ORFs with predicted lengths less than 100 nt were filtered out. We then used CD-HIT software to remove redundant information (protein level) and obtain the initial non-redundant gene catalogue. We selected an identity value of 95% and a coverage value of 90% for clustering, and the longest sequence was selected as the representative sequence. Next, the clean data for each sample were compared with the original gene catalogue using SOAP aligner software, and the number of reads of each gene in each sample was obtained. Those genes in each sample with 2 or fewer reads were filtered out. We then obtained the distribution of reads for the reference genes. Moreover, the abundance information of each gene in each sample was obtained. We obtained a total of 603,031 open reading frames (ORFs). Genes with both start and stop codons accounted for 29.38%~49.66% of the genes in each sample, and genes with neither initiation nor termination codons accounted for 5.83–12.09%. The average ORF length was 347.36 Mbp. The average length for each sample is shown in Table 5.

**Table 5:** The statistical information of predicted gene

Sample	ORFs NO	Integrity: none	Integrity: all	Total length	Average length
MZB.1	39,513	3,004 (7.6%)	19,623 (49.66%)	25.86	654.52
MZB.2	1766	103 (5.83%)	718 (40.66%)	0.5	284.09
MZF.1	57,458	6,949 (12.09%)	16,882 (29.38%)	31.42	546.78
MZF.2	43,961	3,734 (8.49%)	18,541 (42.18%)	26.64	606
MZF.3	27,858	2,972 (10.67%)	10,265 (36.85%)	16.63	597.07
MZF.4	91,228	8,049 (8.82%)	37,356 (40.95%)	54.37	595.93
MZF.5	47,236	3,832 (8.11%)	20,673 (43.77%)	27.95	591.7
MZM.1	67,006	7,037 (10.5%)	24,051 (35.89%)	39.32	586.82
MZM.2	105,623	8,052 (7.62%)	46,010 (43.56%)	54.35	514.59
MZM.3	21,266	2,107 (9.91%)	8,287 (38.97%)	12.07	567.54
MZM.4	50,935	4,540 (8.91%)	20,947 (41.12%)	29.4	577.3
MZS.1	49,063	4,797 (9.78%)	20,141 (41.05%)	28.82	587.47
NOVO_MIX	118	4 (3.39%)	57 (48.31%)	0.03	234.56

### Species abundance

**Fig. 1:**
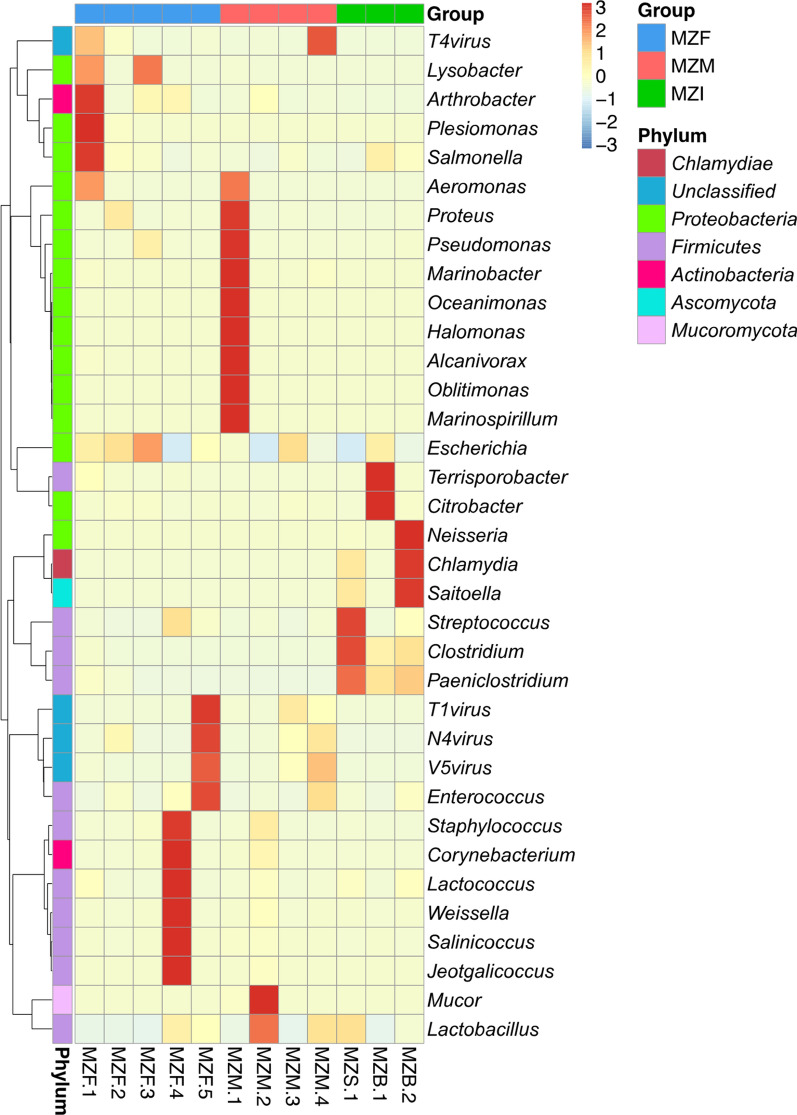
Cluster heat map of relative abundance at genus level

Based on the relative abundance table of different classification levels, the top 35 genera with respect to abundance and their abundance information in each sample were selected to constructed a heat map. Clustering was conducted at the species level to visualize the data and identify the species with higher levels of aggregation in the samples (Fig. 1).

**Fig. 2:**
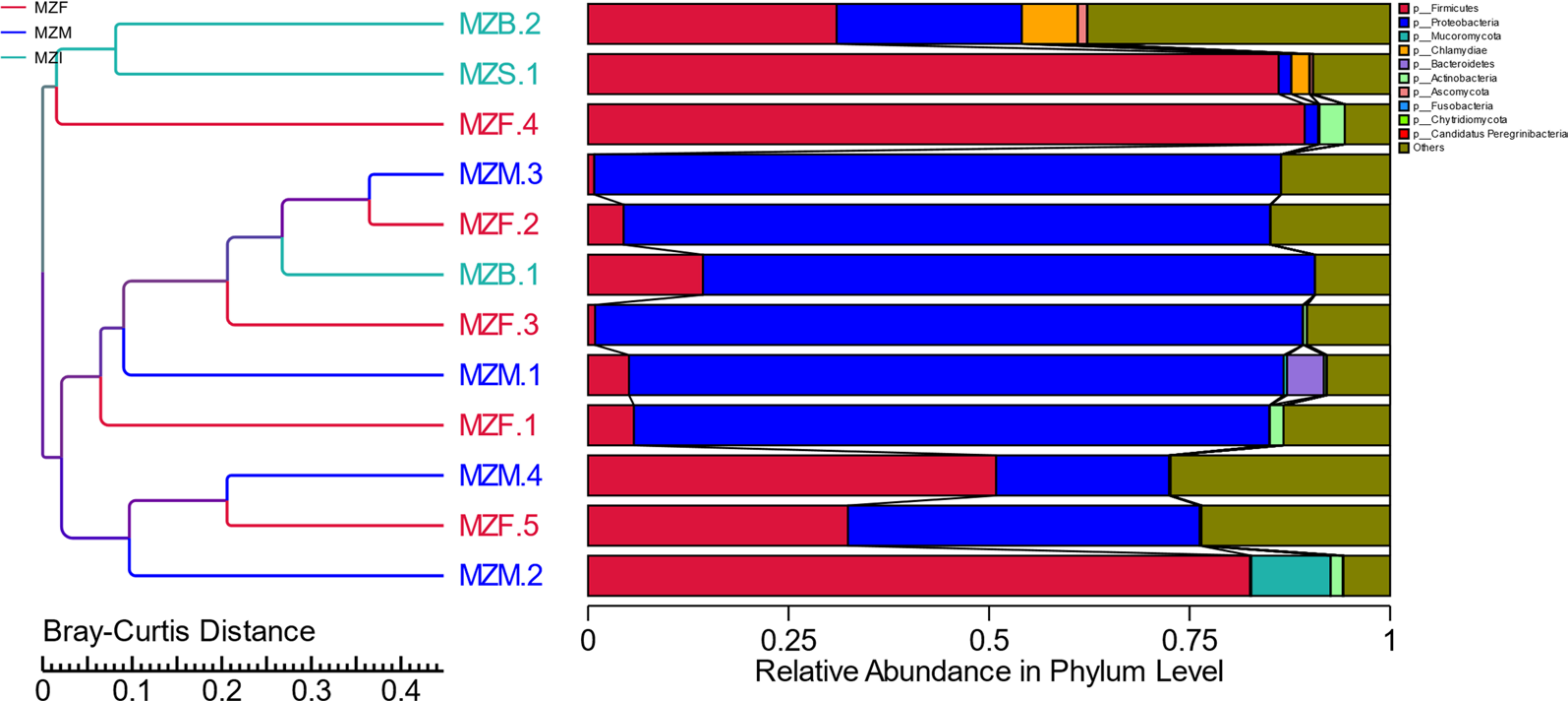
Relative abundance of gut bacterial at the phylum taxonomical level

To visualize the relative abundance data of the intestinal flora, boxplots of the relative abundances of gut bacteria at the phylum level were constructed. Firmicutes and Proteobacteria were the preponderant phyla in all groups (Fig. 2).

### Principal component analysis

**Fig. 3:**
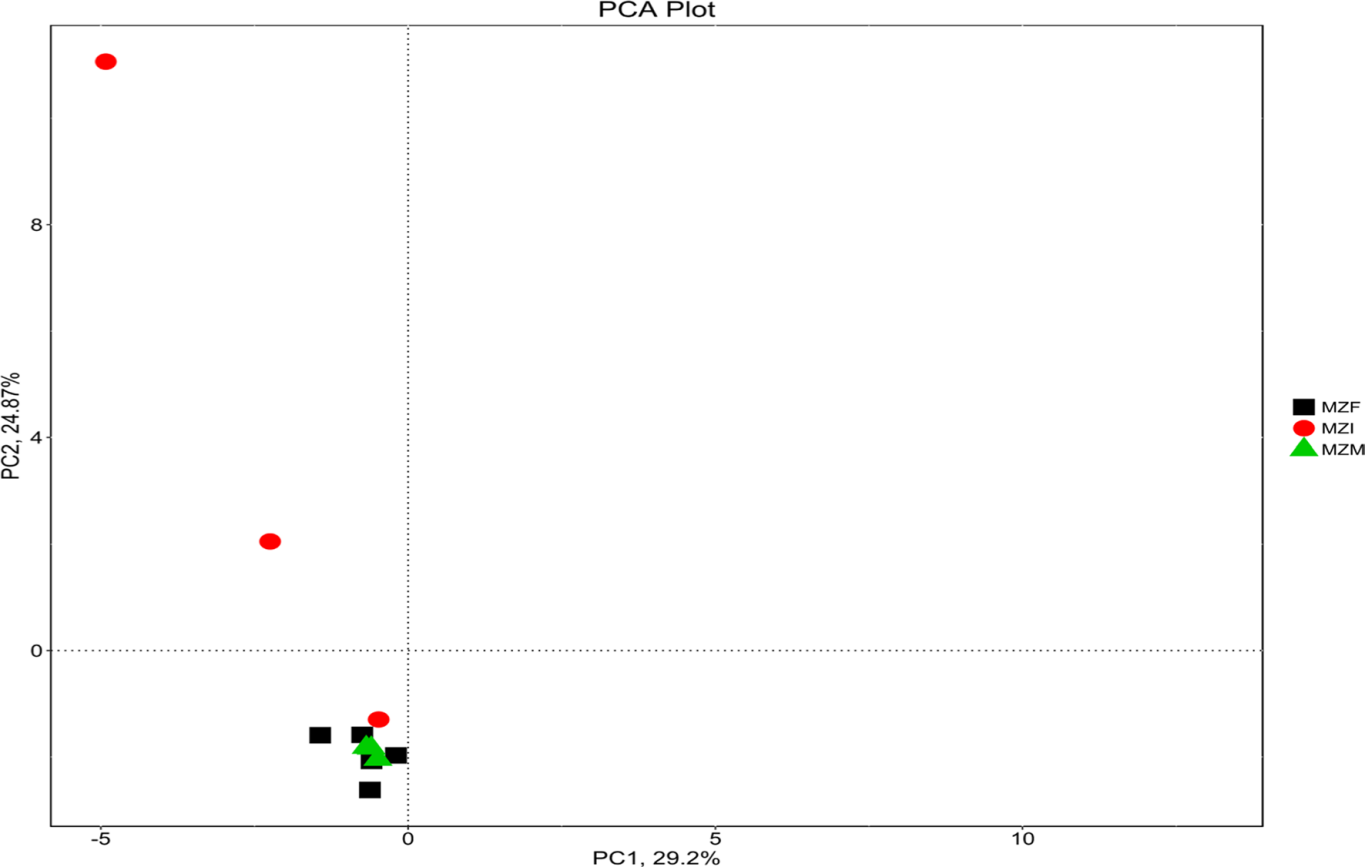
Principal Component Analysis

Because of the complexity of sample data, we used Principal Component Analysis (PCA) to reduce and simplify the sample data. Results from principal component analysis (PCA) are shown in Fig. 3.

### KEGG annotation results

**Fig. 4:**
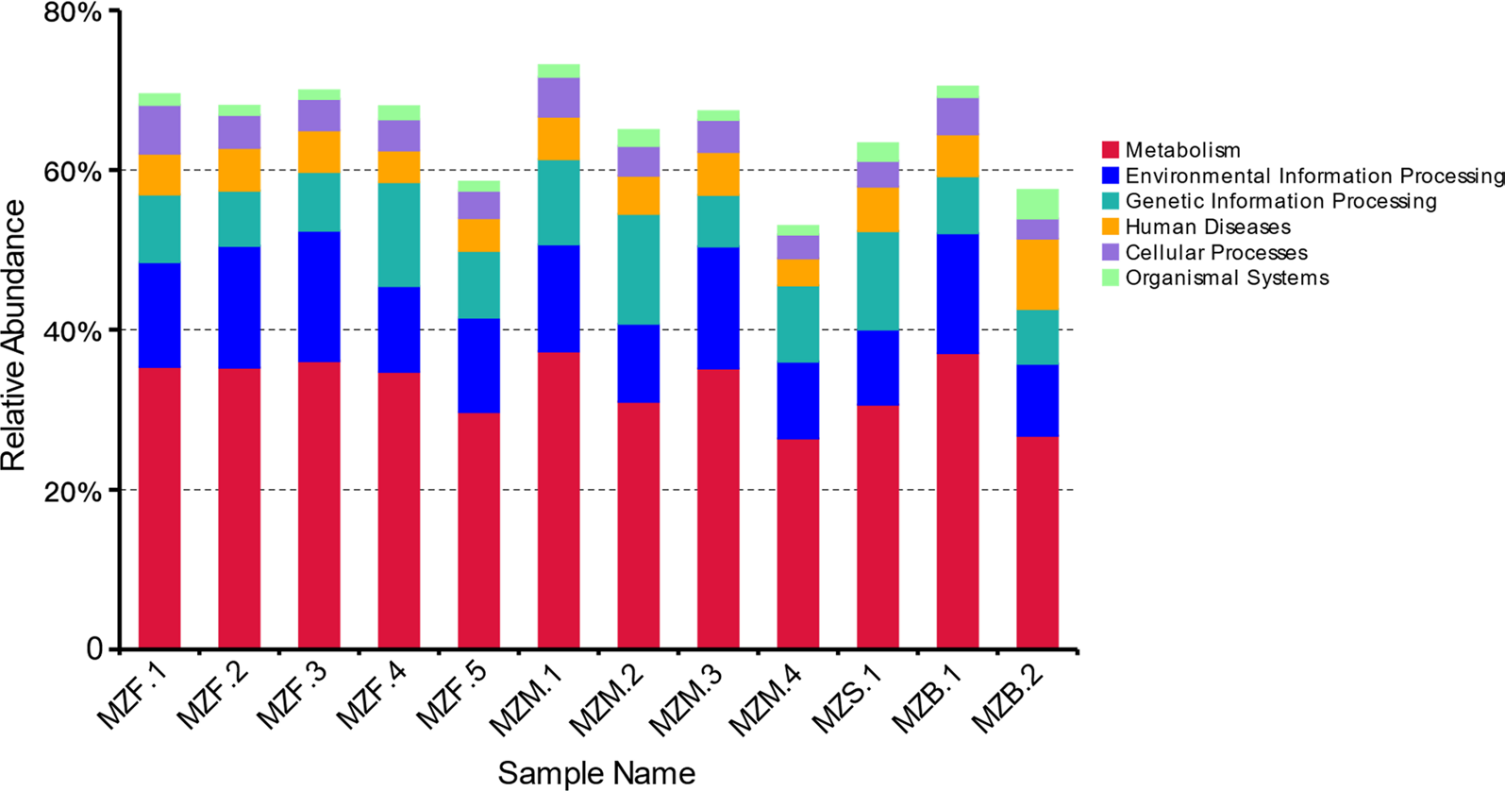
Relative abundance of pathways

**Fig. 5:**
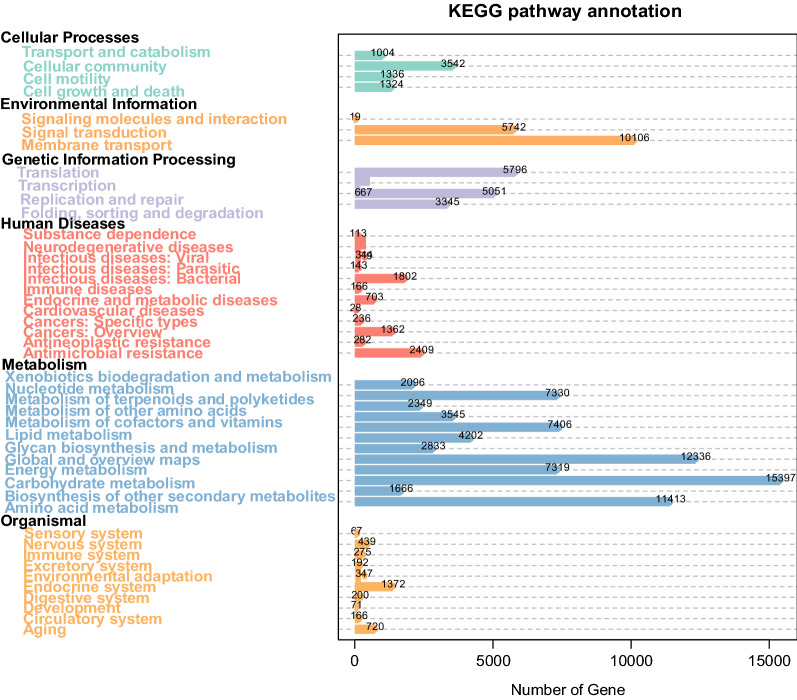
KEGG pathway annotation

The predicted unique genes were searched against the KEGG functional database, and 7140 genes were obtained. The total number of genes across all samples reached 127,839. As shown in Fig. 4, the category associated with the highest number of genes was carbohydrate metabolism, accounting for 11.86% of the genes, which suggests that carbohydrate, as the most important energy supplier, is the main energy source provided to the host by the intestinal flora. Among the processing functions, membrane transportation was associated with a high proportion of genes, accounting for 7.79% of the total number of genes in all samples. This finding indicates that continuous exchange of nutrients and metabolites occurs between the intestinal flora and the host via membrane transportation, with the intestinal microorganisms aiding host digestion of food and providing the host with vitamins and amino acids. Based on the KEGG metabolic pathway annotations, we acquired information on gene function (Fig. 5).

### CAZy annotation results

**Fig. 6:**
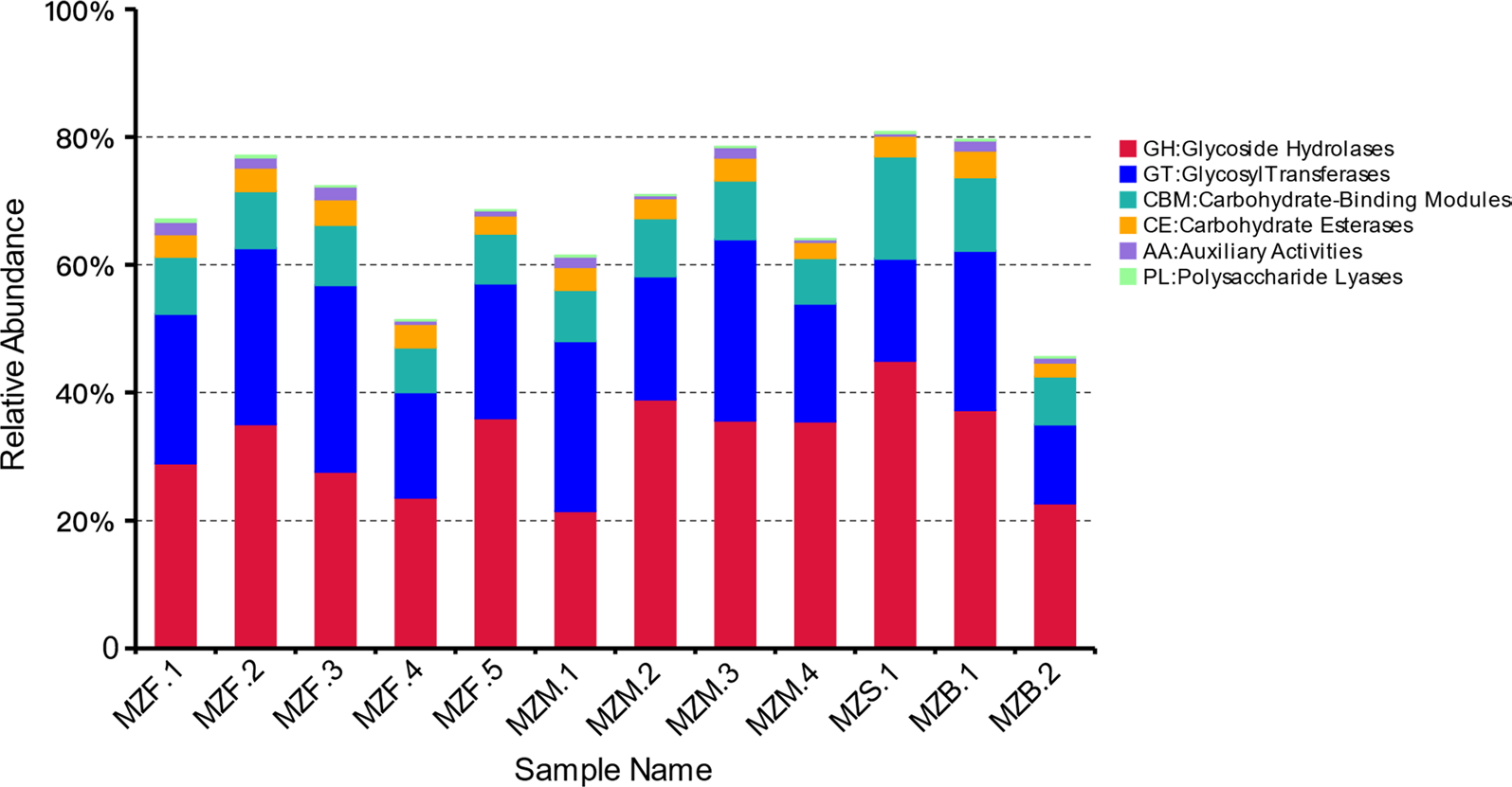
Relative abundance of carbohydrates

**Fig. 7:**
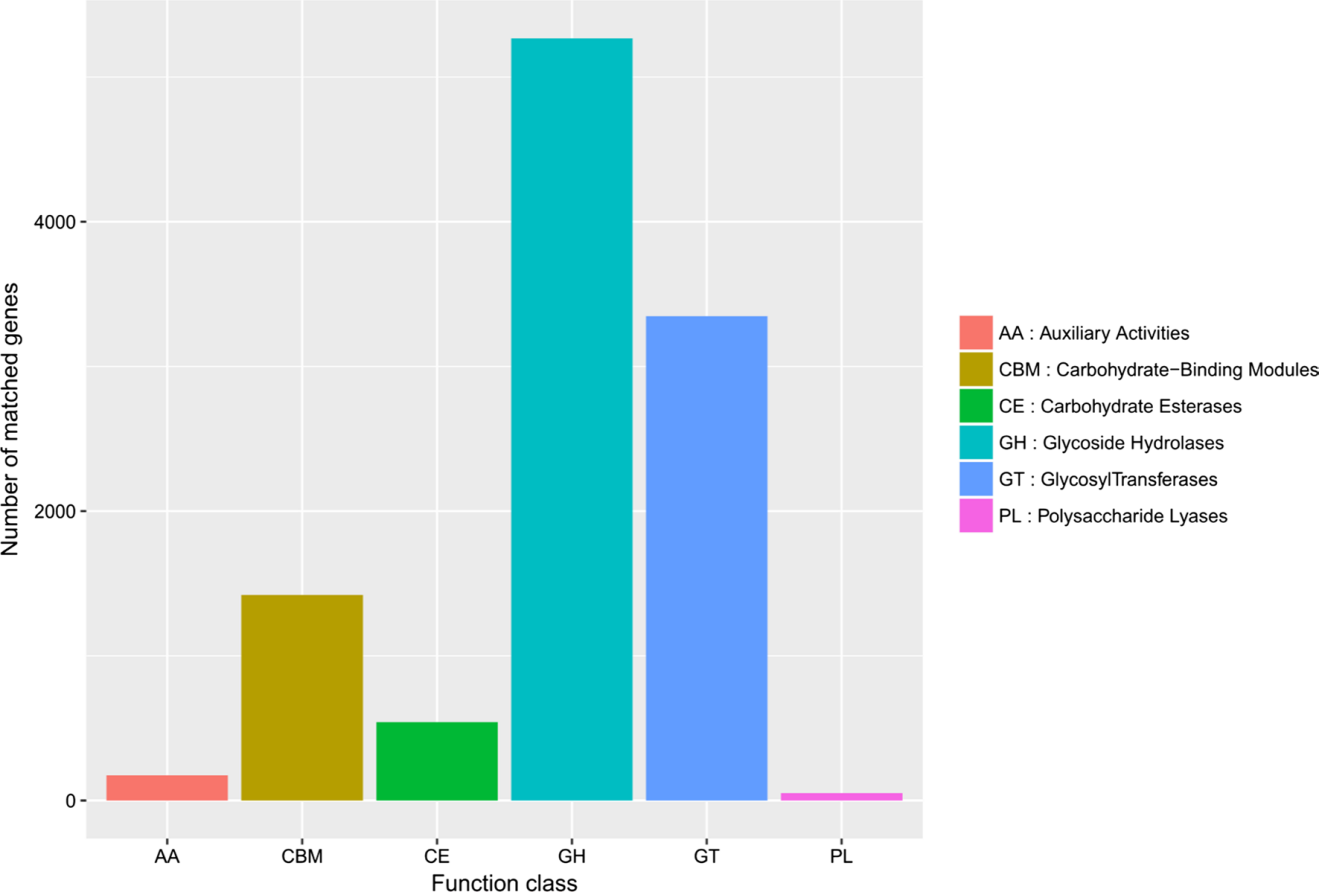
Number of matched genes of carbohydrates

Searching the unique genes against the CAZy database (Carbohydrate-Active Enzymes database), the number of genes corresponding to the six categories of carbohydrate enzymes was obtained. As shown in Fig. 3, the category GH (glycoside hydrolases) corresponded to the greatest proportion of genes, and the category PL (polysaccharide lyases) corresponded to the lowest number of genes. Based on the annotation results, the relative abundances of genes belonging to the six carbohydrate enzyme categories were plotted in a bar chart (Fig. 6), and the numbers of matched genes of carbohydrates were plotted (Fig. 7).

### EggNOG annotates

**Fig. 8:**
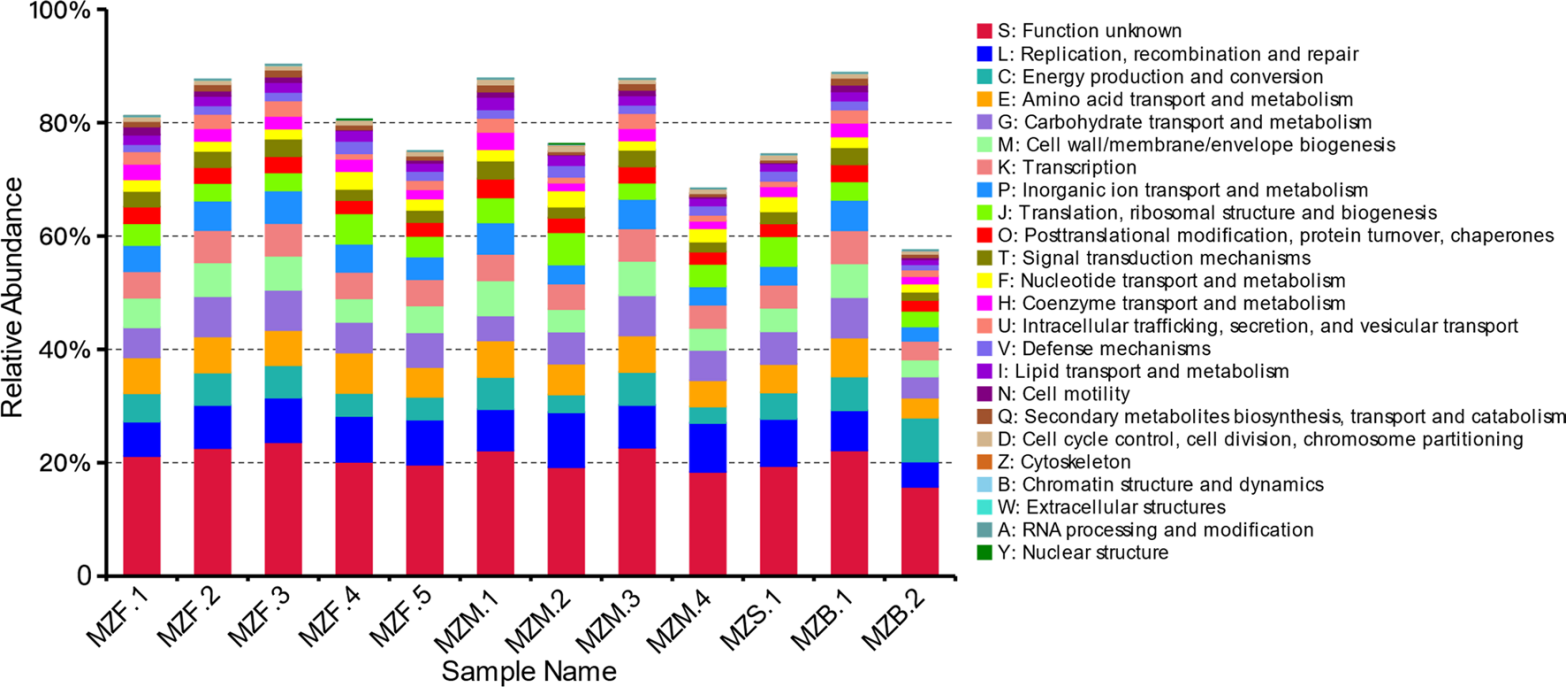
Relative abundance of function class

**Fig. 9:**
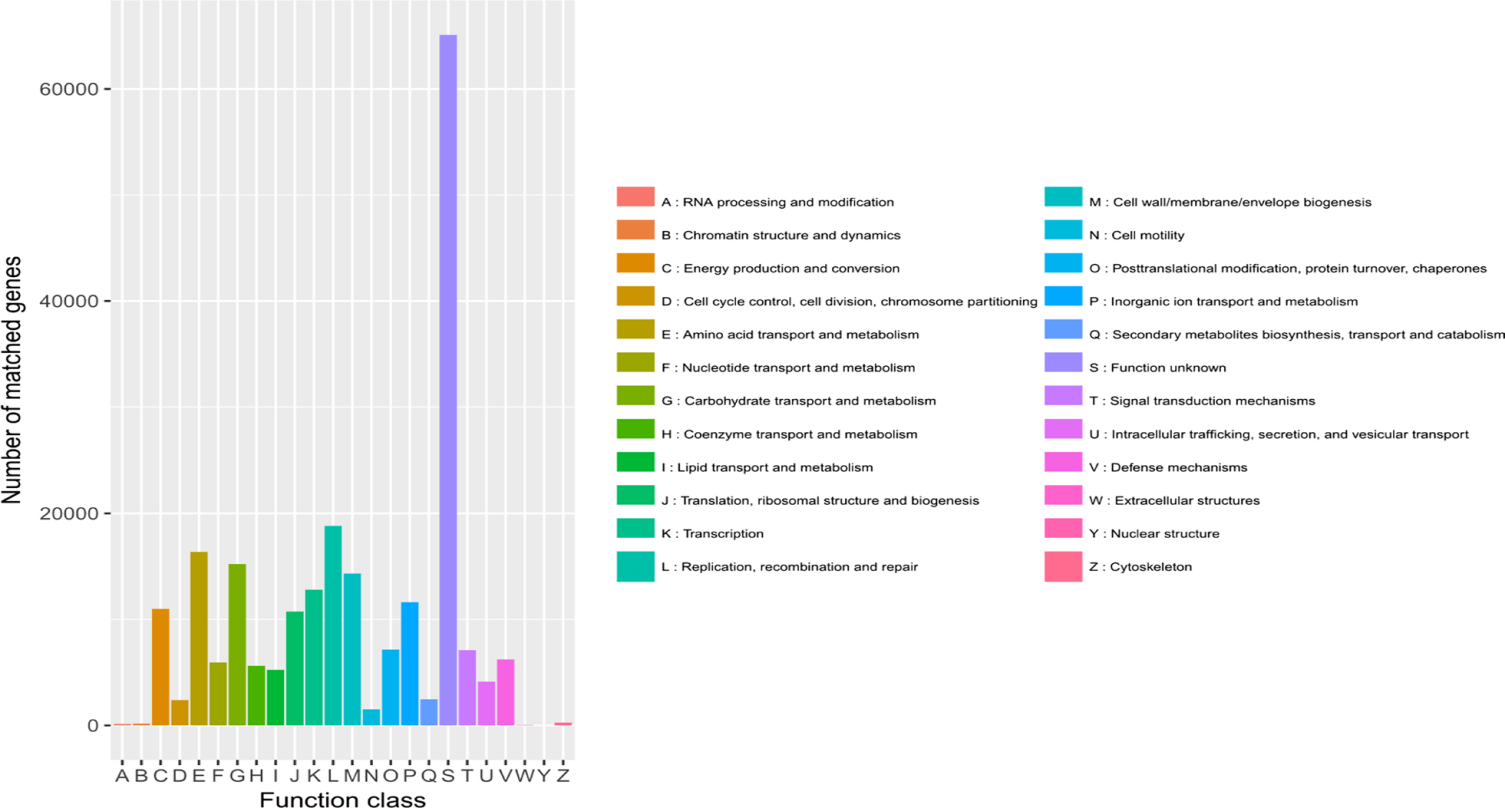
Number of matched genes of function class

The eggNOG database encompasses the COG, KOG and Orthologous Groups databases, and was used to obtain corresponding functional annotations of the sequences. Comparison of the unique genes with genes in the eggNOG database revealed that the main functions of the genes included gene replication, repair of amino acid transport, carbohydrate metabolism and transport. The eggNOG database annotation results are shown in Figs. 8 and 9.

### Resistant gene annotation

**Fig. 10:**
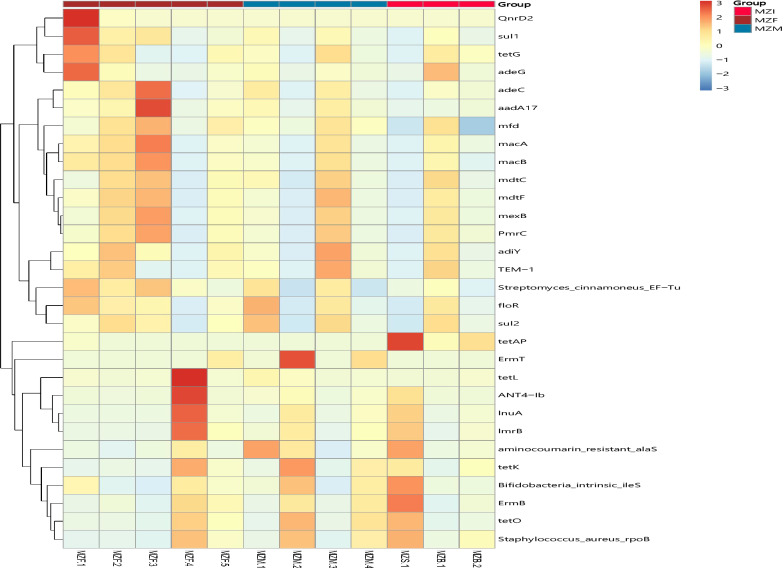
ARO distribution and abundance cluster heat map. The right vertical axis is the ARO name, and the left vertical axis is the ARO cluster tree

**Fig. 11:**
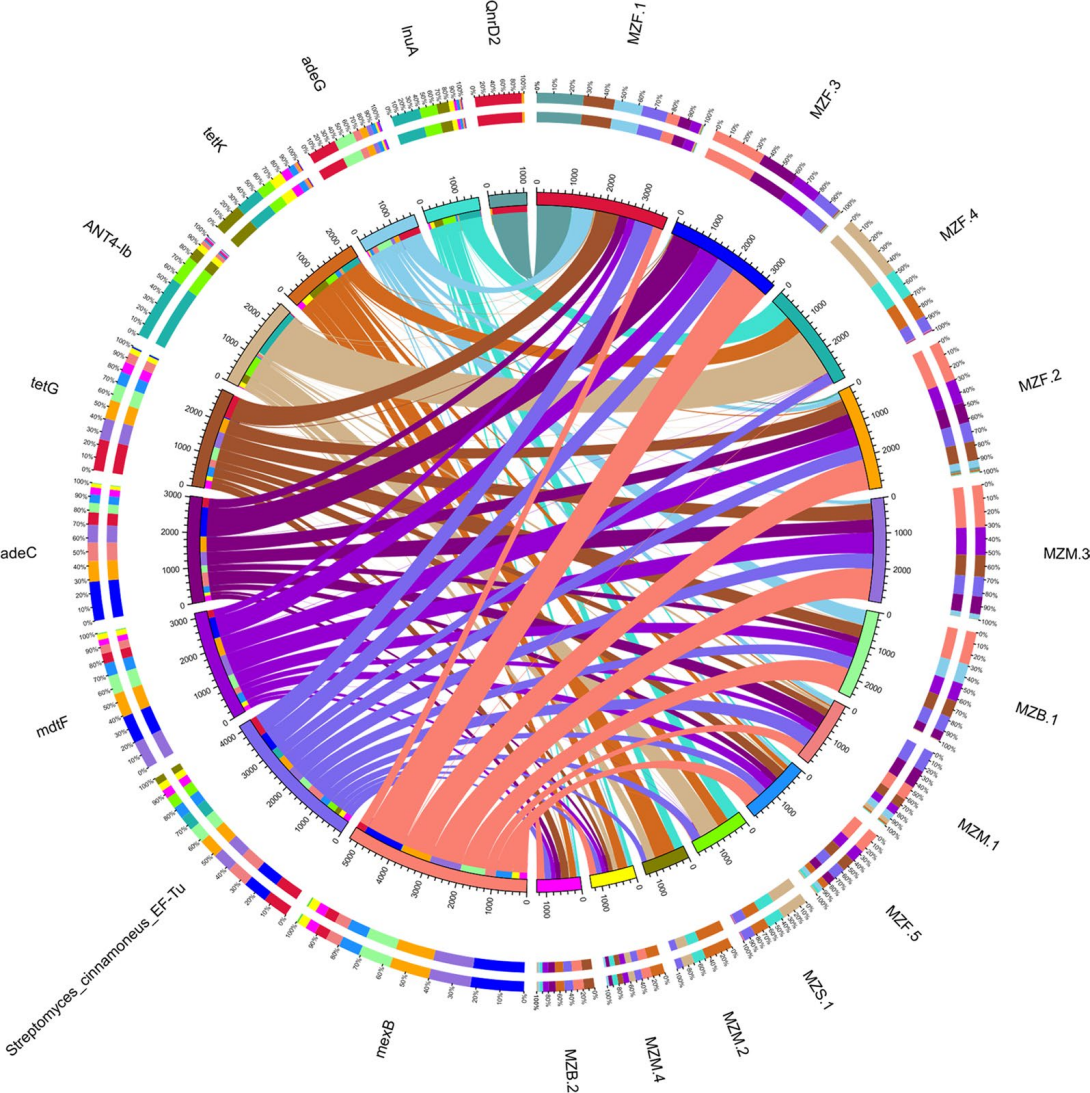
The overview circle graph of resistance gene

To reflect the distribution of ARO in each sample, based on the abundance information of ARO in each sample, the top 30 AROs were selected to construct an abundance cluster heat map (Fig. 10). In addition, based on the annotation results of the CARD database, a network diagram of species associated with resistance genes was constructed (Fig. 11).

## Discussion

The dominant phyla in the human and mouse gut are *Firmicutes* and *Bacteroidetes* (Consortium 2012), and in chickens, the four most abundant bacterial phyla are *Firmicutes*, *Proteobacteria*, *Bacteroidetes* and *Actinobacteria* (Choi et al. 2014). *Firmicutes* and *Bacteroidetes* are the two most prevalent bacteria phyla found in the ruminant gastrointestinal tract (Ye et al. 2016). Among fish, the two most common and abundant bacterial phyla are *Proteobacteria* and *Fusobacteria* (Hill et al. 2016; Wong and Rawls 2012). Regarding invertebrates, *Proteobacteria* and *Firmicutes* have been identified as the dominant phyla in the gut bacterial communities of *Drosophila melanogaster* (Broderick and Lemaitre 2012), and *Proteobacteria* has been identified as the dominant phylum in *E. sinensis* (Chen et al. 2015). The results from this study show that *Proteobacteria* and *Firmicutes* are the dominant bacteria among the intestinal microorganisms of sable at the phylum level. Below the phylum level, the most abundant taxa were similar among the samples, although they differed significantly in abundance. The dominant species within the phyla *Bacteroidetes*, *Tenericutes*, *Proteobacteria* and *Firmicutes* were similar among the samples, demonstrating close relationships of these taxa with the host. Our studies support the view there these microbial taxa have coevolved with their sable host.

The KEGG annotation results revealed that the number of genes corresponding to the function metabolism reached 77,891, representing 60.93% of the total number of genes. Within the metabolism category, the subcategory associated with the highest number of genes, 15,397, was carbohydrate catabolism, which accounted for 11.86% of the genes. These results indicated that the bacterial community of sable is closely associated with digestive tract function. At the order level, *Enterobacteriales*, *Lactobacillales* and *Clostridiales* were the dominant bacterial taxa, demonstrating that the intestinal flora plays significant roles in carbohydrate metabolism. The dominant order of intestinal microorganisms within *Firmicutes* was *Clostridium*, which is involved in the decomposition of cellulose. In addition, some carbohydrate catabolism and vitamin synthesis are usually performed by the intestinal flora (Gao et al. 2020).

In the CAZy database annotation, the number of genes corresponding to glycoside hydrolases was 5267. Glycoside hydrolases are involved in the synthesis of glycoconjugates. The number of glycosyltransferase-associated genes was 3347. The main function of glycosyltransferases is to attach activated sugar groups to various receptor molecules. The number of genes corresponding to carbohydrate-binding modules was 1421, and the number corresponding to carbohydrate esterases was 542. The number of polysaccharide lysate-associated genes was 51, and the number of genes involved in auxiliary activities was 174. These enzymes participate in the degradation and modification of carbohydrates and the formation of glycosidic bonds. Recently, much research has been aimed at determining the complexity of the relationships between the host and the gut microbiota.

Although intestinal microbes are closely associated with the immune system, their relationships with gut immunity remain unknown. The intestinal flora plays important roles in maintaining intestinal health (Zhou et al. 2019). In this study, based on sequence alignment using non-redundant CARD databases and the annotation results, we found an abundance of multiple drug-resistant Mexb protein genes, indicating that the gut microflora plays a role in the immune response to exogenous substances. Regarding the functions of genes in the category environmental information processing, the subcategory membrane transport was associated with the highest number of genes, accounting for 7.79% of the total genes of all samples. This finding indicates that the host and intestinal flora are constantly exchanging substances. The genes associated with the category inorganic transport and metabolism encode phosphate, sulfate, and various cation transporters (Gill et al. 2006). The identified microbial proteins were searched in the COG functional database, which revealed high expression levels of genes associated with inorganic transport and metabolism in healthy children and low levels in obese children with non-alcoholic fatty liver disease (Michail et al. 2014). The cell wall/membrane/envelope biogenesis genes participate in transmembrane transport and the exocytosis of antibiotics to resist the effects of tetracycline hydrochloride, indicating that gut microbiota can enhance antibiotic resistance. Hence, opportunistic microorganisms can survive in the mouse gut (Horie et al. 2017). The numbers of genes related to genetic information processing, such as gene replication, transcription, translation and repair, was 14,859, accounting for 11.62% of the total genes. The annotation results revealed a large number of genes related to host diseases in the intestinal flora; 8167 such genes were identified, accounting for 6.39% of the total. Innate immunity is a significant host defence mechanism that lacks the high selection mechanisms of adaptive immunity. This observation is consistent with our findings. Interestingly, TLRs have been found to be expressed at low levels in the gut of *Drosophila melanogaster* (Broderick and Lemaitre 2012). Many studies have shown that the correlations between microbial community composition and inflammatory parameters can serve as biological indicators of diseases (Becattini et al. 2016). *Lactobacillaceae* and *Enterobacteriaceae*, the dominant bacteria at the family level, play important roles in assisting the host breakdown of carbohydrates and ferment sugars to maintain host nutrition and metabolism. At the genus level, *Lactobacillus* and *Escherichia* were observed at high abundance. *Lactobacillus*, as a beneficial bacterium, act as a barrier against foreign invaders, inhibits the growth of pathogenic bacteria and synthesises vitamins and amino acids for host, maintaining a dynamic balance of gut microbes. This bacterium also plays an important role in tumour inhibition; in some hosts with disease, *Lactobacillus* abundance in the gut is decreased (Azad et al. 2018).

In addition, genes regulating cell processes were identified in the intestinal flora. The number of genes regulating cell growth and apoptosis was 1324, representing 1.04% of the genes. The number of genes regulating cell movement was 1336, representing 1.05% of the total. The number of genes regulating the cell community was 3542, representing 2.77%. The number of genes regulating the transport and catabolism of cells was 1004, representing 79%. The number of genes related to biological systems was 3849, representing 3.01% of the total genes. In addition, in this functional category, the number of genes related to the endocrine system was 1372, accounting for 1.07% of the total.

In this study, we identified the complex population structure of the intestinal microbiota of sables based on metagenomic sequencing methods, which use whole metagenomic data, and we mapped the obtained sequences to known genes or pathways in existing databases, such as CAZy, KEGG, and eggNOG. We then explored the genetic composition and functional diversity of the microbial community based on the mapped functional categories.

## Data Availability

All the raw sequences were submitted to the NCBI Sequence Read Archive, under Accession Number SRP265006.
